# FACTORS ASSOCIATED WITH FATIGUE AMONG PEOPLE WHO HAVE RETURNED TO WORK AFTER STROKE: AN EXPLORATORY STUDY

**DOI:** 10.2340/jrm.v56.18668

**Published:** 2024-03-14

**Authors:** Anna NORLANDER, Ingrid LINDGREN, Christina BROGÅRDH

**Affiliations:** 1 Department of Health Sciences, Faculty of Medicine, Lund University, Lund; 2 Department of Neurology, Rehabilitation Medicine, Memory Disorders and Geriatrics, Skåne University Hospital, Lund, Sweden

**Keywords:** cerebral stroke, cross-sectional studies, fatigue, follow-up studies, regression analysis, return to work, self-report, stroke rehabilitation

## Abstract

**Objective:**

To explore the associations between fatigue impact and (a) personal and stroke-related characteristics, (b) functional impairments and (c) work-related factors among individuals who have returned to work after stroke.

**Design:**

A cross-sectional exploratory study.

**Subjects:**

87 working stroke survivors.

**Methods:**

This study comprises data from a postal survey targeting work ability and perceived stroke-related consequences 1 year after stroke. Fatigue was evaluated using the Fatigue Severity Scale (FSS). Factors associated with having fatigue (FSS total score ≥ 4) were identified using univariable and multivariable logistic regression analyses. Three domain-specific multivariable models and 1 final combined model were created.

**Results:**

Fatigue was reported by 43% of the participants. Several factors representing all the investigated domains were associated with fatigue. In the final combined regression model, self-perceived low cognitive functioning, low decision control at work and high quantitative job demands had the strongest independent effects on the odds of having fatigue.

**Conclusion:**

Among people who were working 1 year after stroke, fatigue was associated with both personal and stroke-related characteristics as well as functional impairments and work-related factors. This highlights the complex nature of post-stroke fatigue. Fatigue management interventions should have a comprehensive approach and also consider the work environment.

LAY ABSTRACT

A total of 87 individuals who returned to work after stroke were studied to explore whether having fatigue (abnormal lack of energy that hinders daily activities) was related to their personal characteristics, to their disabilities following stroke and to their work situation. Postal questionnaires were used to assess their perceived work situation and remaining consequences of stroke, including fatigue, 1 year after stroke. It was found that almost half (43%) of the participants experienced fatigue, and that several personal and stroke-related characteristics, functional impairments and work-related factors were associated with having fatigue. In particular, self-perceived low cognitive functioning, low decision control at work and high quantitative job demands increased the odds of fatigue. This study indicates that post-stroke fatigue is influenced by a combination of factors. This means that helping people manage their fatigue necessitates a holistic approach that considers various aspects, including their work situation.

Stroke is one of the most common causes of complex and long-lasting disability among adults globally (1, 2). In Sweden, more than 25,000 people suffer a stroke every year, of whom approximately 16% are < 65 years (i.e. of working age) (3). A variety of impairments may occur after stroke, including sensorimotor and cognitive impairments as well as fatigue (4–6). Fatigue can be a persistent problem even among those who have otherwise recovered well (7–9). It has been estimated that around 50% of all stroke survivors experience fatigue during the first 2 years after stroke, but prevalence figures vary between 25% and 85% in different study populations (10).

Fatigue is a multidimensional construct with different definitions across studies. It is commonly considered a subjective experience that includes disproportionate mental or physical exhaustion and lack of energy that is triggered by usual activities and does not ameliorate with normal rest (10, 11). The experience of post-stroke fatigue can interact with other cognitive and physical stroke symptoms and depression (12) but is considered a distinct separate phenomenon. Several studies have shown that post-stroke fatigue is related to reduced participation and quality of life (13–15) and can result in difficulties in returning to work (13, 16, 17). It has also been revealed that many of those who do resume working after stroke struggle with problems related to fatigue, which can negatively affect their ability to remain working in the long term (18, 19). We recently showed that the prevalence of fatigue among people who had returned to work within 1 year after stroke was as high as 42%, and that fatigue interfered with work or other responsibilities (20).

Currently, there is no efficient medical treatment to reduce fatigue, which is partly due to a lack of understanding of its underlying causes. Most researchers conclude that the causes of fatigue are complex and multifactorial. It has been proposed that different factors influence early and late fatigue and that some factors may be predisposing whereas others are triggers or perpetuating factors (21, 22). Stroke-specific and biological factors may trigger fatigue in the acute post-stroke phase (i.e. early fatigue) while psychosocial and behavioural factors as well as remaining impairments can contribute to persisting problems (i.e. late fatigue) (8, 21–23). Studies investigating physical, cognitive and psychological factors as well as sleep disturbances and pain have been called for (21).

Moreover, findings from qualitative studies suggest that support from others might play a part in how fatigue is perceived and dealt with (24, 25). A supportive work environment and opportunity to influence the work situation have also been described as important factors to be able to continue working after stroke (26, 27). However, few studies on stroke have investigated factors related to fatigue specifically among working persons, or whether fatigue is associated with factors related to the work situation. Improved knowledge is needed to identify risk factors for persisting fatigue in individuals who are about to return to work after stroke. It is also important to identify potential modifiable factors that can contribute to more sustainable work environments for people with fatigue. Enabling people with stroke to continue working is an important factor for well-being but also to reduce the economic burden on the social welfare system (28).

Thus, the aim of this study was to explore the associations between fatigue impact and personal and stroke-related characteristics, functional impairments and work-related factors among people who have returned to work after stroke.

## METHODS

### Study design

This study is part of a larger project that comprises both quantitative and qualitative data on people who have returned to work after stroke (20, 26, 27, 29). The present study includes a subset of cross-sectional data from a postal survey targeting personal factors, work ability, working situation and perceived consequences of stroke, including fatigue, 1 year after stroke.

### Recruitment of participants

Potential participants were identified through monthly screening of patients admitted to the outpatient stroke rehabilitation clinic at Skåne University Hospital, which is the third largest hospital in Sweden. The uptake area covers 14 municipalities in rural and urban areas. Inclusion criteria were: age 18–64 years, having experienced a stroke (i.e. cerebral infarction, intracerebral haemorr-hage or subarachnoid haemorrhage) 1 year previously (± 2 months), worked at least 25% of full time prior to the stroke, had resumed working after stroke and was still working at the time of the survey. Exclusion criteria were: not being able to answer a questionnaire due to extensive language deficit or cognitive impairment. Written informed consent was obtained from all participants before inclusion in the study. The study was approved by the Regional Ethical Review Board in Lund, Sweden (Dnr 2016/1064) and the principles of the Helsinki Declaration were followed. The collected data (see below) were handled confidentially and securely stored separately from any personal contact information.

### Data collection

The postal survey was carried out between 2017 and 2019. It comprised an invitation letter, a written consent form, questions on demographics (sex, age, living situation) and work situation (return to work, work rate), a set of questionnaires (fatigue, stroke-impact, self-efficacy, psychological and social factors at work), a number of study-specific questions (see below) and a pre-stamped return envelope. Information on stroke type (cerebral infarction or haemorrhage) was retrieved from medical journals.

*Fatigue Severity Scale (FSS).* The FSS is a self-reported fatigue rating scale concerning the perceived impact of fatigue on daily life. The respondent is asked to rate their agreement with statements such as “Fatigue causes frequent problems for me”, “Fatigue prevents sustained physical functioning” or “Fatigue interferes with my work, family or social life”. The 9-item Swedish translated version (30) was used. Each item is scored on a Likert scale ranging from 1 (strongly disagree) to 7 (strongly agree). The total score (range 1–7) is the mean score of the 9 items, where a higher score indicates more fatigue. Most commonly, a cut-off of ≥ 4 is used to classify post-stroke fatigue (10). The FSS was originally developed to assess fatigue in individuals with multiple sclerosis (31). It is also the most frequently used fatigue assessment scale in stroke research (32) and has demonstrated good validity and reliability in this population (33, 34).

*Stroke Impact Scale 3.0. (SIS).* The SIS is a valid and reliable, self-report questionnaire that evaluates disability and health-related quality of life after stroke (35). It includes 59 items in 8 domains. For this study, the 3 domains assessing memory and thinking (7 items), mood and emotions (9 items) and mobility (9 items) were used. The items are scored on a 5-point Likert scale ranging from 1 (could not do it at all) to 5 (not difficult at all). A standardized score ranging from 0 to 100 is calculated for all domains, with higher scores indicating fewer difficulties. In addition, the respondent also rates his/her perceived overall stroke recovery on a visual analogue scale from 0 to 100 where 100 means full recovery (domain 9).

*General Self-Efficacy Scale (GSE).* The GSE assesses the individual’s overall confidence in his or her ability to manage different life situations (36) and has been demonstrated to have good psychometric properties after stroke (37, 38). The scale comprises 10 statements with 4 response categories ranging from 1 (not at all true) to 4 (exactly true). The total sum score ranges between 10 and 40, where a higher score represents better self-efficacy. Although there is no official cut-off score, dichotomization at 30 or around the median is recommended (36).

*General Nordic Questionnaire for Psychological and Social Factors at Work (QPS Nordic).* The QPS Nordic is a comprehensive, valid and reliable questionnaire to assess psychological and social factors at work (39). The original questionnaire comprises 24 subscales, whereof the following 6 were used in this study: Quantitative job demands, Decision control, Work pace control, Support from superior, Support from co-workers and Support from friends and relatives. Each subscale has 2–5 questions with 5 response categories ranging from 1 (very seldom or never) to 5 (very often or always). The total subscale score (range 1–5) is the mean score of the questions in each subscale. On the subscale for quantitative job demands, a higher score means higher demands. For the remaining subscales a higher score means a higher level of control or support. One question regarding decision control (i.e. “Can you decide when to have contact with clients?”) was not applicable to 7 of the participants in the present study. Therefore, this question was excluded when calculating the subscale score for these participants.

*Study-specific questions.* The respondents were also asked about their education level (elementary school/high school/adult further education/university), if they had a sedentary or mobile job (sitting/mobile/both sitting and mobile), if they had received work-oriented rehabilitation after their stroke (yes/no) and if they experienced any pain, sleep disturbance or visual impairment that affected their working ability (yes/no).

### Data analysis

Participant characteristics were reported using descriptive statistics (means and proportions). Factors associated with fatigue 1 year after stroke were identified using univariable and multivariable logistic regression analyses with the dichotomized FSS total score as the dependent variable (i.e., total score ≥ 4 = fatigue, and total score < 4 = no fatigue). Potential explanatory (independent) variables comprised personal and stroke-related characteristics, functional impairments and work-related factors. The selected factors were based on existing research on post-stroke fatigue and on the authors’ own extensive experience from working with people with stroke and fatigue. Before being included in the regression analyses, continuous variables were checked for sufficient linear association with fatigue using the Hosmer– Lemeshow test. For age and self-efficacy, the linear relationship did not hold (i.e. Hosmer–Lemeshow *p* < 0.05), and therefore, these variables were dichotomized. The cut-off values were set close to the median. For self-efficacy, this aligns with the recommended cut-off of 30 on the GSE total score, as suggested by Schwarzer (36). Categorical variables with more than 2 categories (i.e. education and sedentary or mobile job) were also dichotomized to avoid categories with very few respondents.

First, the association with fatigue was evaluated for all the selected variables (*n* = 20) through univariable logistic regression analyses. After this, 3 separate multivariable models were created that represented (a) personal and stroke-related characteristics, (b) functional impairments and (c) work-related factors. An initial generous inclusion criterion of *p* < 0.2 was used for inclusion into multivariable analysis. One variable at a time was then manually removed (starting with the variable with the highest *p*-value) until 4 variables remained in each domain-specific model. To prevent overfitting of the models, a maximum of 4 independent variables per model was allowed based on the current sample size. Before the analysis, the independent variables in each model were checked for multicollinearity and no variables were highly correlated (i.e. Spearman cc < 0.6). All models that included continuous variables were also tested using the Hosmer–Lemeshow test to verify the linearity assumptions of the models.

To identify the variables exhibiting the strongest independent association with fatigue across the domain-specific models, a final combined model that included the most significant variables from all 3 models was constructed. The same procedure as for the preceding multivariable analyses was used, with the exception that 5 independent variables were allowed in the final combined model (see results). With more variables, the risk of overfitting increases, and the results of this model should be interpreted accordingly. *P*-values < 0.05 were considered statistically significant. All statistical analyses were performed using the IBM SPSS Statistics 28/29 software.

## RESULTS

### Participants

**Fig. 1:**
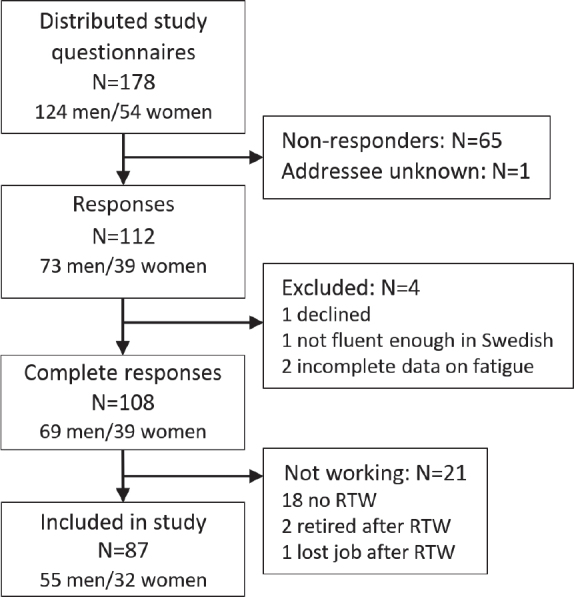
Flowchart showing the inclusion of participants who had returned to work (RTW) after stroke.

Of 178 potential participants, 108 completed the survey. Of these, 87 had returned to work and were still working (Fig. 1). The participants were 55 (63%) men and 32 (37%) women. Their mean age was 53 years (SD 8) and ages ranged from 29 to 65 years. Several reported being almost or fully recovered from their stroke (median 90% recovered, IQR 76–95, on the SIS domain 9). Before stroke, 93% had worked at least 75% of full-time employment. At the time of the survey, 24% reported a lower work rate compared with before the stroke (see Table I). The participants represented a wide range of professions, including blue-collar and white-collar jobs, and had different levels of education and salary levels. Fatigue (i.e. total score ≥ 4 on the FSS) was reported by 37 (43%) of the participants, of whom 23 (26%) had a score of 5 or more, indicating severe fatigue. The mean FSS total score was 3.6 (SD 1.5). Additional and more detailed participant characteristics can be found in Tables I and II.

**Table I:** Participants’ characteristics at stroke onset and one year after stroke, *N* = 87

Age at stroke onset, mean (standard deviation)	52 (8)
Sex, *n* (%)	
Male	55 (63)
Female	32 (37)
Degree of full-time* employment before stroke, *n* (%)	
50–74%	6 (7)
75–100%	81 (93)
Degree of full-time* employment one year after stroke, *n* (%)	
< 50%	10 (12)
50–74%	16 (18)
75–100%	61 (70)
Salary level, *n* (%)	
Low income	4 (5)
Middle income	57 (65)
High income	26 (30)
Self-reported overall recovery one year after stroke (SIS-9), *n* (%)	
30–69% recovered	8 (9)
70–89% recovered	25 (29)
90–100% recovered	54 (62)
Fatigue one year after stroke, *n* (%)	
Yes (FSS total score ≥ 4)	37 (43)
No (FSS total score < 4)	50 (57)

* In Sweden, full-time employment is equivalent to 40 working hours per week. Changes within the categories, e.g. reduction from 100 to 75%, are not known.

Salary level corresponds to full-time (low income: < 20,000 SEK/month, middle income: 20,000–40,000 SEK/month, high income: >40,000 SEK/month); SIS-9: Stroke Impact Scale (version 3.0) domain 9; FSS: Fatigue Severity Scale (total score range 0–7).

**Table II:** Description of the independent variables and their association with fatigue (dichotomized FSS score) one year after stroke based on univariable regression analyses, *N* = 87

Independent variables and response categories*	Score distribution** *n* (%) / Md (q1-q3)	Univariable regression Odds ratio (CI)	*p*-value
PERSONAL AND STROKE-RELATED CHARACTERISTICS			
** Sex**			
** **Women	32 (37)	3.00 (1.22–7.41)	***0.017***
** **Men (ref.)	55 (63)		
** Age at time of survey**			
** **< 50 years	28 (32)	1.94 (0.78–4.83)	***0.154***
** **≥ 50 years (ref.)	59 (68)		
** Living alone**			
** **Yes	18 (21)	1.99 (0.68–5.55)	0.214
** **No (ref.)	69 (79)		
** Self-efficacy (GSE)** ^a^			
** **Low: total score 10–30 p	29 (34)	3.46 (1.35–8.81)	***0.009***
** **High: total score 31–40 p (ref.)	56 (66)		
** Education level**			
** **Has a university degree, min. 3 years	34 (39)	1.65 (0.69–3.95)	0.260
** **No university degree (ref.)	53 (61)		
** Stroke type**			
** **Cerebral infarction, CI	69 (79)	3.21 (0.96–10.73)	***0.058***
** **Cerebral haemorrhage, ICH/SAH (ref.)	18 (21)		
FUNCTIONAL IMPAIRMENTS			
** Memory & thinking (SIS domain 2)**^b^ Domain score, range 0–100	93 (79–100)	0.91 (0.87–0.95)	***< 0.001***
** Mood & emotions (SIS domain 3)**^b^ Domain score, range 0–100	78 (61–89)	0.941 (0.912–0.971)	***< 0.001***
** Mobility (SIS domain 6)** Domain score, range 0–100	100 (97–100)	0.909 (0.825–1001)	***0.052***
** Pain**			
** **Yes	12 (14)	5.036 (1.257–20.175)	***0.022***
** **No (ref.)	75 (86)		
** Sleep disturbance**			
** **Yes	11 (13)	7.714 (1.555–38.266)	***0.012***
** **No (ref.)	76 (87)		
** Visual impairment** ^b^			
** **Yes	13 (15)	3.616 (1.017–12.860)	***0.047***
** **No (ref.)	73 (85)		
WORK-RELATED FACTORS			
** Sedentary or mobile job**			
** **Only sitting	30 (34.5)	1.959 (0.799–4.803)	***0.142***
** **Mobile or partly mobile (ref.)	57 (65.5)		
** Work rehabilitation** ^b^			
** **Have not received work rehabilitation	37 (43)	1.496 (0.631–3.548)	0.361
** **Have received work rehabilitation (ref.)	49 (57)		
** Quantitative job demands (QPS N.)**^b^ Subscale score, range 1–5	2.5 (2.0–3.1)	1.831 (1.070–3.132)	***0.027***
** Work pace control (QPS N.)**^b^ Subscale score, range 1–5	3.8 (2.8–4.3)	0.704 (0.446–1.113)	***0.133***
** Decision control (QPS N.)**^b^ Subscale score, range 1–5	3.4 (2.8–4.2)	0.444 (0.260–0.756)	***0.003***
** Support from superior (QPS N.)**^a^ Subscale score, range 1–5	4.0 (3.0–5.0)	0.553 (0.352–0.870)	***0.010***
** Support from co-workers (QPS N.)** Subscale score, range 1–5	4.0 (4.0–5.0)	0.532 (0.309–0.916)	***0.023***
** Support from friends/relatives (QPS N.)** Subscale score, range 1–5	4.0 (3.3–5.0)	0.542 (0.345–0.853)	***0.008***

* For categorical variables, the response category used as reference in the univariable regression is marked with (ref.).

** *n* (%) for categorical values and md (q1–q3) for continuous variables.

a = 2 missing,

b = 1 missing.

FSS: Fatigue Severity Scale (total score cut-off for fatigue ≥ 4); GSE: General Self-Efficacy Scale; SIS: Stroke Impact Scale (domain score 0–100, higher score means less disability); QPS N: General Nordic Questionnaire for Psychological and Social Factors at Work (note: for the “quantitative job demands” subscale a higher score means higher demands. For the remaining a higher score means a higher level of control or support).

Associations with *p* < 0.2 (i.e. variables that qualified for inclusion in multivariable analysis) are highlighted in bold italics.

### Factors associated with fatigue after return to work

*Personal and stroke-related characteristics.* The univariable analyses revealed that female sex and low self-efficacy were significantly associated with having fatigue. Age below 50 years and having a cerebral infarction had slightly weaker associations with fatigue (Table II). These 4 variables remained independently associated with fatigue in the multivariable analysis, with stroke type and self-efficacy demonstrating the highest odds ratios (Table III). The odds of having fatigue were 11 times higher if having a cerebral infarction compared with a cerebral haemorrhage and 9 times higher if having low self-efficacy compared with high self-efficacy.

**Table III:** Results of the multivariable regression models for (a) personal and stroke-related characteristics, (b) functional impairments and (c) work-related factors, with fatigue as the dependent variable

Independent variables	Odds ratio (95% CI)	*p*-value	Nagelkerke R-square
PERSONAL AND STROKE-RELATED CHARACTERISTICS			
Stroke type (cerebral infarction vs ref. haemorrhage)	11.02 (2.16–56.22)	***0.004***	0.362
Self-efficacy, GSE (low vs ref. high)	9.28 (2.58–33.33)	***< 0.001***	
Age (< 50 years vs ref. ≥ 50 years)	4.22 (1.35–13.16)	***0.013***	
Sex (women vs ref. men)	3.51 (1.20–10.27)	***0.022***	
FUNCTIONAL IMPAIRMENTS			
Memory & thinking, SIS domain 2 (0–100)	0.93 (0.89–0.98)	***0.004***	0.436
Mood & emotions, SIS domain 3 (0–100)	0.96 (0.93–1.00)	*0.051*	
Sleep disturbance (yes vs ref. no)	3.09 (0.47–20.39)	0.241	
Visual impairment (yes vs ref. no)	2.52 (0.55–11.51)	0.232	
WORK-RELATED FACTORS			
Decision control, QPS N. subscale (1–5)	0.514 (0.26–1.00)	*0.051*	0.289
Quantitative job demands, QPS N. subscale (1–5)	2.02 (1.06–3.84)	***0.033***	
Support from friends/relatives, QPS N. subscale (1–5)	0.72 (0.41–1.22)	0.222	
Support from superior, QPS N. subscale (1–5)	0.83 (0.47–1.47)	0.521	

Fatigue: Dichotomized Fatigue Severity Scale total score (cut-off ≥ 4); GSE: General Self-Efficacy Scale (low = total score 10–30, high = total score 31–40); SIS: Stroke Impact Scale (domain score 0–100, higher score means less disability); QPS N: General Nordic Questionnaire for Psychological and Social Factors at Work (subscale score 1–5, higher score means higher control/demands); associations with *p* < 0.05 are highlighted in bold italics.

*Functional impairments.* In the univariable analyses, higher scores (i.e. better functioning) on the SIS memory & thinking and the SIS mood & emotions domains were significantly associated with lower odds of having fatigue, whereas experiencing pain, sleep disturbance and visual impairment were associated with higher odds of fatigue (Table II). These 5 variables, as well as SIS mobility, were initially entered into the multivariable analysis. Only memory & thinking remained significantly associated with fatigue and mood & emotions was very close to being significant with a *p*-value of 0.051 (Table III).

*Work-related factors.* In the univariable analyses, higher quantitative job demands were significantly associated with higher odds of fatigue, whereas higher decision control and higher support from superiors, co-workers and friends/relatives were associated with lower odds of fatigue. Work pace control and sedentary or mobile job had weaker associations with fatigue (Table II) but were also included in the multivariable analysis. Only quantitative job demands remained significantly associated with fatigue in the multivariable model and decision control was very close to significant (Table III).

*Final combined model.* The 6 variables that were significantly associated with fatigue in the 3 multivariable models (i.e. stroke type, self-efficacy, age, sex, memory & thinking and quantitative job demands) as well as the 2 variables that were very close to significance (i.e. mood & emotions and decision control) were included in a combined model. The analysis revealed that better memory & thinking and higher decision control at work were significantly associated with lower odds of fatigue, whereas higher quantitative job demands increased the odds of fatigue. There was also a tendency that being female and having a cerebral infarction increased the odds of fatigue. In addition to the 4 most significant variables, stroke type was kept in the final model as this variable exhibited a relatively large effect on the odds of fatigue, influenced the other estimates and had a fairly low *p*-value (Table IV). The final combined model had an R-square of 0.5.

**Table IV:** Results of the final combined multivariable regression model, with fatigue as the dependent variable

Independent variables	Odds ratio (95% CI)	*p*-value	Nagelkerke R-square
Sex (women vs ref. men)	3.58 (1.00–12.79)	0.050	0.535
Stroke type (cerebral infarction vs ref. haemorrhage)	5.49 (0.93–32.25)	0.060	
Memory & thinking, SIS domain 2 (0–100)	0.91 (0.86–0.96)	***< 0.001***	
Decision control, QPS N. subscale (1–5)	0.39 (0.17–0.87)	***0.021***	
Quantitative job demands, QPS N. subscale (1–5)	2.18 (1.07–4.43)	***0.031***	

Fatigue: Dichotomized Fatigue Severity Scale total score (cut-off ≥ 4); SIS: Stroke Impact Scale (domain score 0–100, higher score means less disability); QPS N: General Nordic Questionnaire for Psychological and Social Factors at Work (subscale score 1–5, higher score means higher control/demands); associations with *p* < 0.05 are highlighted in bold italics.

## DISCUSSION

Our results showed that personal and stroke-related characteristics as well as functional impairments and work-related factors influenced the odds of having self-reported fatigue among people who were working 1 year after stroke. When factors from all 3 domains were included in a combined model, perceived cognitive impairments and high strain at work appeared to be most strongly associated with fatigue. However, the fact that factors from all domains influenced the model indicates that a combination of factors are likely to impact on the level of fatigue upon work resumption.

The finding that work-related factors were associated with fatigue adds new and important knowledge. Moreover, it should be considered that several of these factors are potentially modifiable. The work-related factors that demonstrated the strongest independent association with fatigue were perceived quantitative job demands and perceived decision control. As assessed by the QPS Nordic, high quantitative job demands entailed having too much work, work that piles up, a high work pace or having to work overtime. Decision control meant having influence over important work-related decisions and control over workload, methods of execution or choice of collaborators. Whereas higher job demands increased the odds of fatigue, higher decision control lowered the odds of fatigue. Occupational health research has shown that an imbalance between perceived work demands and perceived control notably affects mental well-being. In particular, a combination of high demands and low control contributes to heightened psychological strain (40). Despite the lack of previous studies specifically investigating work-related factors in relation to post-stroke fatigue, findings from qualitative research show that high work demands as well as low control are perceived as barriers for retaining work among individuals with post-stroke fatigue (18, 19, 27). These studies also indicate that a gradual return to work, with reduced or flexible hours or duties and appropriate adjustments to the workplace, can facilitate reintegration into a sustainable working life. The fact that support from neither superiors nor co-workers or friends/relatives remained significant in our multivariable analyses was somewhat surprising as several studies have demonstrated that social support, especially from superiors, is a key factor for successful return to work after stroke (19, 41). However, a majority of our participants reported having relatively good social support, and it may play a greater role in enabling return to work than in influencing levels of fatigue. While larger and prospective studies are necessary to corroborate our findings, our results coupled with insights from interviews with stroke survivors underscore the importance of addressing the work situation when dealing with post-stroke fatigue.

Concerning personal and stroke-related characteristics, all factors in the domain-specific multivariable model were associated with fatigue. The association between fatigue and female sex is consistent with previous research (6, 42). Regarding age, previous results are inconsistent (22) but it has been proposed that a higher perceived impact of fatigue among younger individuals could be attributed to more demanding daily responsibilities or social roles (43). The finding that cerebral infarction increased the odds of having fatigue is in contrast to previous research showing a higher prevalence of fatigue after cerebral hemorrhage (32). An explanation could be that our participants were younger compared with the overall stroke population and slightly more women had suffered an infarction. The result is interesting as there might be different underlying factors for ischemic and haemorrhagic stroke, especially at a younger age, that could be relevant for the development of fatigue. In addition, fatigue was associated with general self-efficacy (i.e. confidence in one’s ability to manage different situations in life). This aligns with other studies which have demonstrated that psychological factors may influence the experience and sustainment of fatigue (11, 22), and that improved self-efficacy may help mitigate fatigue over time (44). It should also be considered that experiencing stroke-related impairments and fatigue could contribute to lower self-efficacy. However, in the final combined model, none of the personal or stroke-related factors remained strongly significant, and only sex and stroke type appeared to directly influence the model.

Turning to functional impairments, the multivariable analysis showed that problems with memory and thinking were most strongly associated with having fatigue. Associations between fatigue and impaired cognitive function have previously been demonstrated (11, 22). Even though the majority of our participants reported only minor impairments, it could be assumed that even discrete cognitive impairments can contribute to increased effort in performing various tasks, thus leading to the experience of fatigue. Memory and concentration difficulties are also considered to be symptoms of mental fatigue. In addition, symptoms overlap with those for mood disorders/depression and an association between fatigue and mood has previously been well-established. Fatigue and depression may interact to a large degree and experiencing fatigue could also affect the mood (45). It was therefore important to include mood as a possible confounder in the analysis. Although SIS mood & emotions had a tendency towards significance in the domain-specific multivariable model, it did not remain in the final combined model.

In terms of clinical implications, this study suggests that targeted interventions, such as cognitive-behavioural therapy or workplace accommodations, aimed at enhancing perceived control and balancing demands might facilitate successful reintegration and sustained employment among individuals with post-stroke fatigue. Additionally, addressing cognitive functioning and emotional reactions is likely an important component of successful fatigue management. Multidisciplinary interventions that encompass these aspects should be considered for this population. Notably, younger women with cerebral infarction may be a particularly vulnerable group that require specific attention.

### Strengths and limitations

Because the data were cross-sectional, we cannot assume causal relationships or their direction. Thus, prospective longitudinal studies that further investigate fatigue in relation to work are warranted. The well-defined study population, with a specific focus on people who have returned to work, is a strength. Not the least considering that the large variation in fatigue prevalence between studies has largely been attributed to differences in study samples and means of assessment (10). Assessing fatigue with the FSS, which is the most common fatigue assessment scale in stroke research, facilitated comparison with other studies. However, the FSS rests on the respondent’s own perception of fatigue and does not distinguish between different types of fatigue. We have previously demonstrated that different fatigue rating scales may capture different aspects of fatigue (20). It is therefore possible that other associated factors might have been identified if fatigue were assessed in a different way. Most of the investigated factors were self-rated and can reflect a respondent’s proneness to different response styles. On the other hand, the individual’s own perception of his or her situation is likely to be important for how well-being is affected.

The survey had a response rate of 62%. Even though that is normal for this type of follow-up, the lack of information concerning the non-responders is a limitation. Other studies have shown that non-responders are typically male, of lower socioeconomic status and have poorer mental and physical health compared with responders. People with busy schedules are also less likely to participate in research (46). The fact that this study targeted people in mid-life who had returned to work after stroke may have contributed to several people not responding.

The limited sample size may have led us to identify (i.e. achieve significance for) only the factors that were most strongly associated with fatigue. Also, dichotomizing some variables may have resulted in the loss of certain information. However, given the relatively unexplored nature of this field, identifying key factors is an important foundation for future work. A benefit of the multivariable analyses is that they indicate which variables have the most robust independent association with fatigue. This does not mean that the other factors, especially those that were close to significant, are not of importance.

### Conclusion

Among people who were working 1 year after stroke, fatigue was associated with both personal and stroke-related characteristics as well as functional impairments and work-related factors. This highlights the complex nature of post-stroke fatigue. Fatigue management interventions should have a comprehensive approach and also consider the work environment.
